# Preoperative and Postoperative Patient-Reported Outcome Measurement Information System Scores in Patients Treated for Benign Versus Malignant Soft Tissue Tumors

**DOI:** 10.7759/cureus.25534

**Published:** 2022-05-31

**Authors:** Jonathan F Dalton, Ryan Furdock, Landon Cluts, Bharadwaj Jilakara, Douglas Mcdonald, Ryan Calfee, Cara Cipriano

**Affiliations:** 1 Department of Orthopaedic Surgery, University of Pittsburgh Medical Center, Pittsburgh, USA; 2 Orthopaedic Surgery, University Hospitals Cleveland Medical Center, Cleveland, USA; 3 Orthopaedics, University of Missouri Kansas City School of Medicine, Kansas City, USA; 4 Orthopaedics, Washington University School of Medicine, St. Louis, USA; 5 Orthopaedics, University of Pennsylvania Perelman School of Medicine, Philadelphia, USA

**Keywords:** orthopaedic oncology surgery, promis, soft tissue tumors, patient reported outcomes, sarcoma

## Abstract

Background: The Patient-Reported Outcomes Measurement Information System (PROMIS) assesses multiple aspects of patient well-being but has not been thoroughly studied amongst orthopedic oncology patients.

Questions/purposes: How do preoperative/early postoperative PROMIS scores compare in patients with benign versus malignant soft tissue tumors (STTs) for (1) physical function, (2) pain interference, and (3) depression? Are the differences clinically relevant? What other patient/tumor factors impact PROMIS?

Methods: This retrospective cohort study included 314 STT patients who underwent resection of a benign (n = 187) or malignant (n = 127) STT over a period of 4.25 years at a single institution. PROMIS physical function, pain interference, and depression scores were collected preoperatively and at two and six weeks postoperatively. Scores for each time point were compared between groups and to preoperative baselines. Backward-stepwise linear mixed-effects models were produced to identify independent predictors of change in each PROMIS domain. The minimal clinically important difference (MCID) was 4 points.

Results: The malignant cohort, but not the benign cohort, demonstrated clinically relevant worsening of physical function postoperatively. Malignant diagnosis (△ = −4.4, p < 0.001) and lower extremity tumors (△ = −4.5, p < 0.001) were identified as clinically relevant, independent predictors of worse physical function at all time points. No predictors of clinically relevant changes in pain interference or depression scores, including malignancy, were identified.

Conclusions: In STT patients, malignancy and lower extremity STT location are associated with clinically relevant worsening in physical function but do not significantly impact pain interference or depression in the early postoperative period. These findings may help establish the utility of PROMIS in an orthopedic oncology population.

## Introduction

Anticipating patient outcomes is critical for medical decision-making, preoperative counseling, and postoperative care. Historically, outcome measures have been largely objective and physician-reported, but recently, patient-reported outcome measures have been increasingly used in clinical practice and research [1]. The Patient-Reported Outcomes Measurement Information System (PROMIS) is a computer-adaptive test (CAT) developed by the National Institutes of Health to address deficiencies in traditional fixed-length assessment systems [2]. PROMIS utilizes a series of probability-based computer algorithms to provide the minimum number of informative questions while still achieving high measurement precision [2]. Furthermore, in addition to assessing physical function, PROMIS has domains for pain interference, depression, anxiety, fatigue, and role functioning, which are designed to capture a broader concept of well-being that incorporates elements of mental and social health [1,2]. There is currently a widespread initiative for the adoption and inclusion of PROMIS domains in Food and Drug Administration (FDA)- and industry-funded clinical oncology outcomes research [3,4].

PROMIS has been shown to be sensitive to changes in physical function, pain interference, and depression in studies that expected effects in these domains [5-7]. It has also been found to correlate highly with traditional patient-reported outcomes measurement systems, with the added benefits of reduced floor and ceiling effects, increased reliability, and greatly reduced test length [8-11]. PROMIS has performed well in a variety of orthopedic specialties, including hand, shoulder, foot and ankle, sports, and trauma [9,12-14]. For example, Chen et al. demonstrated that PROMIS scores significantly improved in patients undergoing ACL reconstruction and have the ability to predict postoperative outcomes [13]. However, the ability of PROMIS to detect differences in patient-reported outcomes in the setting of adult sarcoma has only recently been examined in a small number of studies [15-16]. Additionally, no studies have been performed comparing pre- and postsurgical PROMIS scores in patients with benign versus malignant soft tissue tumors (STTs). Such information would provide context for PROMIS scores gathered for these groups in the perioperative period.

The purpose of this study was to examine perioperative changes and outcomes for patients undergoing resection of benign versus malignant STTs. The first hypothesis was that PROMIS physical function, pain interference, and depression domain scores would be significantly different between patients with malignant versus benign STTs. The second hypothesis was that PROMIS would be able to detect changes in these domains over the perioperative period and that the magnitude of change would be clinically relevant. The third hypothesis was that patient- and tumor-specific factors (e.g., age, sex, STT location, and STT diameter) would impact these PROMIS domains. The goal of this project was to provide information to help providers, researchers, and patients interpret PROMIS scores in the setting of STTs as well as add to the limited body of literature validating PROMIS scores in this population [15,17].

## Materials and methods

This retrospective cohort study included patients over 18 years of age who underwent resection of an STT by the Orthopedic Oncology Division at Washington University School of Medicine in St. Louis and was approved by IRB ID 201604100 named "PROMIS and Orthopaedic Oncology." An a priori power analysis was performed to calculate the appropriate number of study participants. Considering the minimal clinically important difference (MCID) published for these PROMIS assessments in oncology patients and in a recent study of patients undergoing surgery for lower extremity metastases, we designed our study to detect a difference of 4 points on each PROMIS CAT between groups [18]. Expecting a 10-point standard deviation on these scales and setting the alpha at 0.05 with a power of 0.80, we calculated that a total of 196 patients would be required to demonstrate a statistically significant difference between the two groups. Patients were included over a 4.25-year period from July 2015 until October 2019.

Medical records were reviewed for patient age and gender, histologic diagnosis, tumor size, and tumor location (axial/pelvic vs. appendicular, upper vs. lower extremity). Computer-adaptive PROMIS surveys were administered on tablet computers (Apple, Cupertino, CA). Patients were asked to complete the PROMIS physical function, pain interference, and depression domains at their preoperative, two-week postoperative, and six-week postoperative clinic appointments. All PROMIS measures were scored with a theoretical range of 0-100 and were scaled to have the normal United States population average of 50 (SD ± 10 points). Higher scores always indicate more of the domain being tested; thus, a score of 60 for physical function indicates a better than average functionality, while a score of 60 on pain interference indicates a greater impact of pain on life. Patients were aware of their diagnoses (i.e., benign versus malignant STT) at the time of their preoperative visit. Patients with benign tumors that had made full recovery (defined as complete wound healing, resolution of symptoms, return to full activity, and discontinuation of pain medication) by two weeks postoperative were only asked to return for a six-week follow-up if they experienced any change in their status. In these situations (n = 132), the two-week scores were also used as six-week scores if the patient did not return.

A total of 394 patients underwent STT resection during the enrollment period. Of these, 46 patients were excluded for surgical treatment that required bone resection or amputation, and 16 were excluded for not having completed the PROMIS surveys during their office visits. An additional 18 patients were excluded due to a lack of follow-up. Of the remaining 314 patients, 187 had benign and 127 had malignant STTs, as diagnosed by histopathologic analysis.

Statistical analysis

A Pearson’s chi-squared test was performed to compare the benign and malignant STT groups for the categorical variables of sex, axial/pelvic versus appendicular location, upper extremity versus lower extremity location, and tumor size greater than 5 cm versus less than 5 cm. ANOVA and two-tailed, unpaired t-tests were used to compare preoperative versus postoperative PROMIS scores within each cohort and to make univariate comparisons between the cohorts. Pearson’s correlation was performed to assess whether changes in the scores of one PROMIS domain were associated with changes in other PROMIS domains. Backward-stepwise, repeated measures mixed models were used to detect the independent effects of demographic variables and time since surgery on each PROMIS domain. All seven collected variables were eligible for inclusion in the model, and variables with a p-value greater than 0.1 were excluded by the stepwise procedure. In all statistical analyses, p-values less than 0.05 were considered significant.

## Results

**Figure 1:**
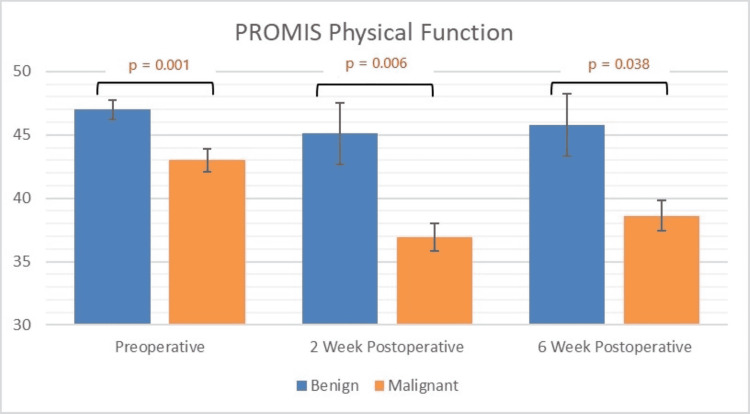
Graphical representation of PROMIS scores for physical function Significant p-values indicated in red

Table 1 presents comparisons of demographic and tumor information between the malignant and benign STT cohorts. Physical function was significantly worse in patients with malignant tumors compared to those with benign tumors at all time points (p < 0.05; Figure 1 and Table 2). The malignant cohort had a significantly worse physical function at two weeks (p < 0.001) and six weeks (p = 0.005) postoperatively compared to their preoperative baselines. The average change in physical function for malignant patients at both two weeks (∆ = −6.1) and six weeks (∆ = −4.4) postoperatively was greater than the MCID of 4 points established for this study (Table 3). Additionally, while physical function scores improved between two and six weeks after surgery for patients with benign STTs, we observed no significant improvement in the malignant STT cohort (p =0.28). Independent predictors of worsened physical function are listed in Table 2. Of these, only malignant STT diagnosis (∆ = −4.4, p < 0.001) and upper extremity tumor location (∆ = 4.5, p < 0.001) exceeded MCID cutoff values.

**Table 1:** Comparisons of benign vs. malignant cohort demographic information Values are presented as mean ± SD or N (column %). p-values: ^a^ANOVA, ^b^Pearson's chi-square test.

Factor		Total (N=314)	Benign STT (N=186)	Malignant STT (N=128)	p-value
Age		54.7±18.7	50.7±18.0	60.5±18.1	<0.001^a^
Sex	Female	161 (51%)	112 (60%)	49 (38%)	<0.001^b^
	Male	153 (49%)	74 (40%)	79 (62%)	
Tumor location	Lower extremity	225 (72%)	130 (70%)	95 (74%)	0.40^b^
	Upper extremity	89 (28%)	56 (30%)	33 (26%)	
	Appendicular skeleton	280 (89%)	176 (95%)	104 (81%)	<0.001^b^
	Axial skeleton	34 (11%)	10 (5%)	24 (19%)	
Tumor size	Tumor diameter (cm)	8.3±6.0	7.7±5.4	9.1±6.6	0.050^a^
	Tumor diameter >5 cm	130 (41%)	87 (47%)	43 (34%)	0.020^b^
	Tumor diameter <5 cm	184 (59%)	99 (53%)	85 (66%)	

**Table 2:** Results of backward-stepwise, repeated-measures mixed models STT: soft tissue tumor

Physical function			
Independent predictors	Effect size (change in physical function score)	95% confidence interval	p-value
Time since surgery	−1.93	−2.5 to −1.3	<0.001
Age (years)	−0.1	−0.03 to −0.2	<0.001
Male sex	2.4	0.3–4.4	0.03
Malignant tumor	−4.4	−6.6 to −2.3	<0.001
Upper extremity	4.5	2.3–6.8	<0.001
Pain interference			
Independent predictors	Effect size (change in pain interference score)	95% confidence interval	p-value
Time since surgery	1.2	0.6–1.8	<0.001
Upper extremity	−3.2	−5.4 to −1.0	0.003
Tumor diameter (cm)	0.2	0.04 to 0.4	0.02
Depression			
Independent predictors	Effect size (change in depression score)	95% confidence interval	p-value
Time since surgery	−0.6	−1.1 to −0.2	<0.001
Upper extremity STT	−2.8	−5.1 to −0.5	0.001

**Table 3:** Mean PROMIS results for physical function, pain interference, and depression domains for benign and malignant STT cohorts across perioperative time points The numbers indicated in bold are those that reached that minimal clinically important difference of 4.

	Physical function				Pain interference				Depression			
Perioperative time	Benign	Standard error	Malignant	Standard error	Benign	Standard error	Malignant	Standard error	Benign	Standard error	Malignant	Standard error
Preoperative	47.0	0.79	43.0	0.93	55.1	0.79	54.8	0.93	46.2	0.72	46.8	0.9
2 week postoperative	45.1	2.43	36.9	1.08	58.2	0.74	60.2	0.88	44.9	0.84	46.8	0.9
6 week postoperative	45.8	2.44	38.6	1.20	56.9	0.77	58.1	0.97	44.4	0.85	46.8	1.09
Change from preoperative vs. 2 weeks postoperative	−1.9		−6.1		3.2		5.3		−1.3		0.0	
Change from preoperative vs. 6 weeks postoperative	−1.2		−4.4		1.8		3.2					

**Figure 2:**
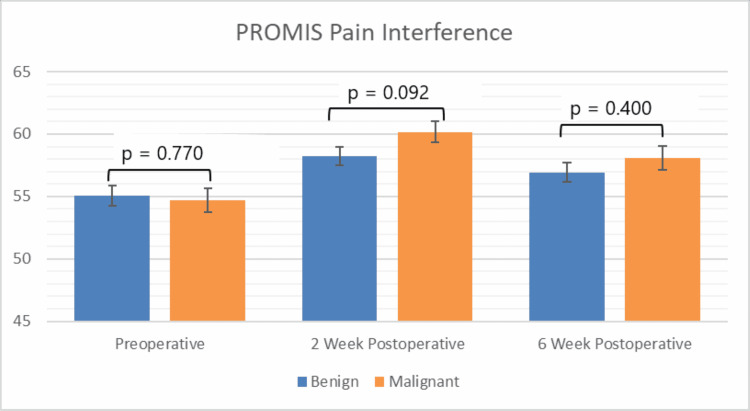
Graphical representation of pain interference PROMIS scores

Interference scores did not differ significantly between patients with benign tumors compared to patients with malignant tumors at any time point (Figure 2 and Table 2). In patients with benign tumors, pain interference was significantly increased two weeks postoperatively compared to the preoperative baseline (p = 0.003). Pain interference scores recovered to preoperative levels by six weeks in the benign STT cohort (p = 0.08). In patients with malignant tumors, both two- and six-week postoperative pain interference scores were significantly higher (indicating worse pain) than the preoperative baseline (p < 0.05). Time since surgery (∆ = 1.2 at 2 weeks, ∆ = 2.4 at 6 weeks, p < 0.001), upper extremity tumor location (∆ = −3.2, p = 0.003), and tumor diameter (∆ = 0.2/cm, p = 0.02) were identified as independent predictors of change in pain interference scores (Table 2). However, none of these factors exceeded the MCID cutoff.

**Figure 3:**
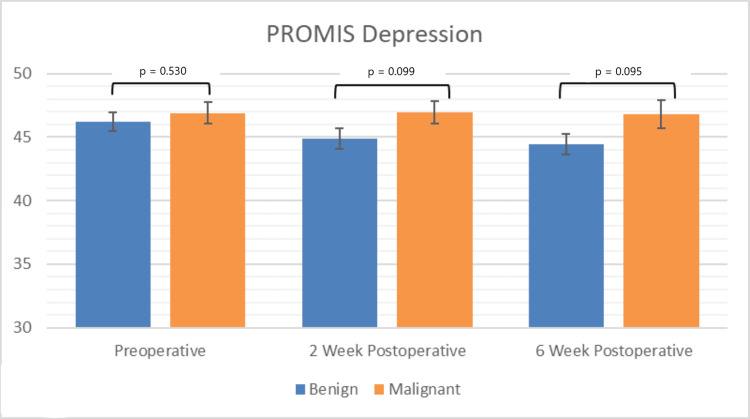
Graphical representation of depression PROMIS scores

Depression scores remained similar between benign and malignant diagnoses across all time points (p > 0.05; Figure 3). Neither the benign nor malignant cohorts exhibited significant variation in PROMIS depression scores over time (p > 0.05). Considering all patients, PROMIS depression scores improved by a mean of 3.77 points postoperatively (p = 0.032). Upper extremity STT location (∆ = 2.8, p = 0.001) and increased time since surgery (∆ = 0.6 at 2 weeks, ∆ = 1.2 at 6 weeks, p < 0.001) independently predicted improved depression scores (Table 2). However, none of these factors exceeded the MCID cutoff.

Increased depression was associated with increasing pain interference preoperatively (r = 0.53, p < 0.001), two weeks postoperatively (r = 0.60, p < 0.001), and six weeks postoperatively (r = 0.63, p < 0.001). Increased depression was also associated with decreased physical function preoperatively (r = −0.41, p < 0.001), two weeks postoperatively (r = −0.34, p = 0.001), and six weeks postoperatively (r = −0.045, p < 0.001). Similarly, increased pain interference was associated with decreased physical function preoperatively (r = −0.64, p < 0.001), two weeks postoperatively (r = −0.63, p < 0.001), and six weeks postoperatively (r = −0.69, p < 0.001).

## Discussion

The malignant STT cohort differed from the benign STT cohort in terms of increased age, male predominance, increased percentage of tumors affecting the axial skeleton, increased overall tumor diameter, and increased percentage of tumors greater than 5 cm. Of these factors, only age and male sex were noted to be independent predictors of change in any of the measured PROMIS domains. Neither of these factors met the MCID cutoff. Despite the differences in age between our cohorts, our malignant STT group reflects the age distribution of patients with STTs in a recent, large epidemiologic study, so our findings are likely generalizable to this population [19]. Finally, tumor diameter was found to be an independent predictor of pain interference, but this also did not meet the MCID cutoff.

In this study, PROMIS was able to detect significant differences in physical function in patients with malignant tumors compared to those with benign tumors at all time points. Additionally, compared to their preoperative scores, patients with malignant STTs experienced decreases in physical function that exceeded MCID cutoffs at both two and six weeks postoperatively, whereas physical function changes in patients with benign STTs did not exceed the MCID cutoff at either time point. While pain interference scores in patients with benign tumors recovered by six weeks postoperatively, patients with malignant tumors did not exhibit similar recovery. Variations in each of the three PROMIS domains studied were associated with changes in the other two domains at each of the perioperative time points measured, consistent with the existing orthopedic literature [20-22].

Physical function domain

Several studies have shown that PROMIS effectively measures changes in physical function during the 6-week postoperative period [23,24]. In our population, PROMIS detected differences in physical function experienced by patients with malignant STTs both postoperatively compared to their preoperative baselines and compared to patients with benign STTs. Factors identified as independent predictors of decreased physical function included malignant diagnosis and older age. Of these variables, only malignant STT diagnosis exceeded MCID cutoffs. The more aggressive operations that are required to achieve negative margins and the adverse physiologic effects of chemotherapy and radiation may have contributed to worsening the physical function associated with a malignant diagnosis [25]. Additionally, quality of life research has indicated that a diagnosis of malignancy takes a significant emotional, social, and physical toll [26]. Notably, prior work has emphasized that the rareness of STS has hindered research into the specific impact that STS have on patient well-being, which highlights the importance of preliminary work such as this [27]. These data may help patients, families, and employers understand that physical function recovery may be prolonged and incomplete.

Pain interference domain

Preoperative pain interference scores were not significantly different in the benign compared to malignant STT cohorts (p = 0.77). This is consistent with the principle that sarcomas most often present as painless masses. While pain interference scores were elevated two weeks postoperatively in all STT patients, they returned to their preoperative baseline by six weeks in the benign cohort but not in the malignant cohort. The increase in pain interference at two weeks postoperatively exceeded MCID cutoffs for the malignant cohort only. This suggests that the more aggressive, complex surgeries needed to excise malignant tumors may cause more pain in the postoperative period, even without increased pain in the preoperative period. Additionally, because no benign STTs received pre- or postoperative radiation, it was not possible to control for the effect of this variable in our analysis comparing benign versus malignant STTs. Presumably, the effect of radiation would impact pain and likely play a role in driving the difference noted in PROMIS pain interference scores. Increasing time since surgery was found to increase pain interference, albeit by a non-clinically significant amount. This is likely a result of our relatively short postoperative follow-up. These results may have implications for setting patient expectations and managing postoperative analgesia.

Depression domain

PROMIS depression scores were similar in patients with benign and malignant STTs across all time points. The multivariate analysis demonstrated significantly lower depression scores (improved depressive symptoms) in patients with upper extremity tumors compared to lower extremity tumors. It is possible that patients with lower extremity STTs experienced more severe depression due to their clinically significant increase in functional impairment postoperatively (e.g., difficulty ambulating). Development or exacerbation of depression in the setting of postoperative lower extremity weakness is consistent with current literature linking worse performance on tests of lower extremity physical function (e.g., the 8-foot up and go test, the 30-second chair stand test) to worsening of depressive symptoms [28]. Time since surgery was also found to independently predict lower depression scores, which would be expected following resection of a tumor.

Limitations

This study has several limitations. Because most benign STTs do not require longer follow up, it includes only the early postoperative period. Thus, our results do not capture intermediate or long-term effects on PROMIS scores. Future goals for this project would be to obtain longer term follow-up with this patient population, which could enable detection of latent effects beyond the perioperative period. Second, our benign and malignant STT groups differed significantly in terms of age, sex, tumor diameter, and tumor location (axial versus appendicular). Multivariable analyses did not demonstrate that patient sex, tumor diameter, or axial versus appendicular location significantly affected PROMIS outcomes. We found increased age to be predictive of poorer outcomes on physical function scores, so this could be a confounding factor for the univariate analyses presented. However, age did not meet the MCID cutoff established for this project. Additionally, the data for this project did not include an assessment of tumor depth, which may have an impact on PROs. However, prior large, multivariate analyses have noted that the potential prognostic value of STT depth can be explained by deeper-seated tumors tending to have a larger mean size, which is captured by this project [29,30].

## Conclusions

PROMIS can detect changes in physical function, pain interference, and depression during early follow-up after STT excision. Patients with malignant STTs are at a greater risk for increased functional deficits and may have greater pain postoperatively. While PROMIS depression scores were higher in patients with lower extremities than in upper extremity STTs, depression scores improved in both groups following the removal of the tumor. Additional research on these and other PROMIS domains is needed to explore the clinical relevance of our findings, determine the long-term outcomes of surgery, and further define the utility of PROMIS in the orthopedic oncology population.
